# Supplementary material for: Ancestral Absence of Electron Transport Chains in Patescibacteria and DPANN
Source: Front Microbiol. 2020 Aug 17;11:1848. doi: 10.3389/fmicb.2020.01848 (PMC7507113; doi:10.3389/fmicb.2020.01848)
Supplement: TABLE S4 — Symbiont genome assemblies and taxonomic names used in Figures 6, 7. [file Table_4.PDF]

IMG Genome identifier	Phylum	Taxonomic name
2512564067	Proteobacteria	Rickettsia montanensis
2568526683	Proteobacteria	Holospora obtusa
2576861681	Proteobacteria	Candidatus Xenolissoclinum pacificiensis
2609460328	Proteobacteria	Candidatus Hepatobacter penaei
2616645016	Proteobacteria	Candidatus Endoecteinascidia frumentensis
2630968813	Bacteroidetes	Candidatus Sulcia muelleri
2636415932	Bacteroidetes	Cardinium endosymbiont cEper1
2740892203	Spirochaetes	Treponema endosymbiont D11
637000036	Spirochaetes	Borrelia burgdorferi
637000331	Actinobacteria	Tropheryma whipplei
642555114	Bacteroidetes	Candidatus Amoebophilus asiaticus
642555127	Elusimicrobia	Elusimicrobium minutum
642555145	Proteobacteria	Orientia tsutsugamushi
646564518	Bacteroidetes	Sulcia muelleri
648028014	Bacteroidetes	Sulcia muelleri
650716011	Bacteroidetes	Blattabacterium sp. Bge
638154511	DPANN	Nanoarchaeum equitans
