# Supplementary material for: Ancestral Absence of Electron Transport Chains in Patescibacteria and DPANN
Source: Front Microbiol. 2020 Aug 17;11:1848. doi: 10.3389/fmicb.2020.01848 (PMC7507113; doi:10.3389/fmicb.2020.01848)
Supplement: DATA S1 — Source files for maximum likelihood phylogenetic trees of concatenated single copy proteins from Bacteria and Archaea (Figure 2). [file UNI56_archaea_v2_mafftBMGE.concat.FTLG.pdf › mdmII_arcbactree_Aug2019/UNI56_archaea_v2_mafftBMGE.concat.FTLG.pdf]

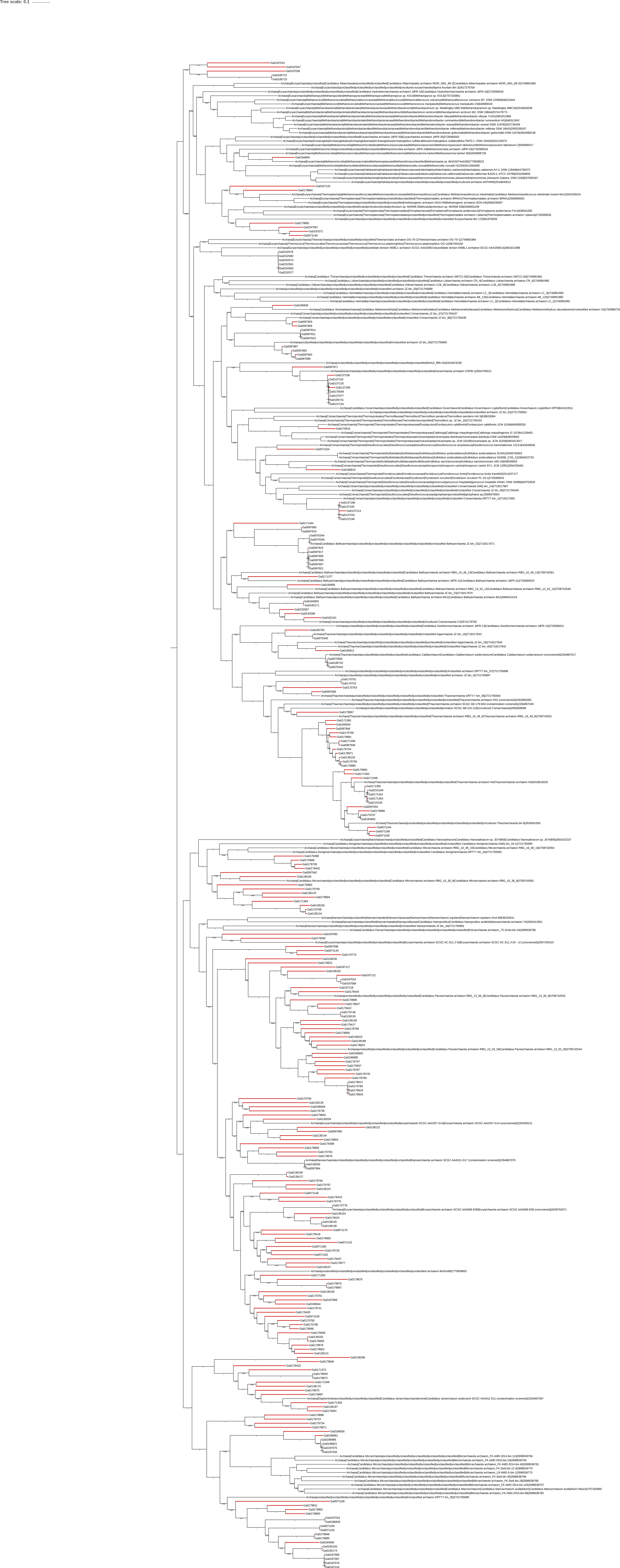
Tree scale: 0.1
